# A comparative analysis of topic modelling techniques for the thematic analysis of student feedback

**DOI:** 10.1371/journal.pone.0328697

**Published:** 2026-08-13

**Authors:** Neha Kardam, Denise Wilson

**Affiliations:** Electrical and Computer Engineering, University of Washington, Seattle, Washington, United States of America; Islamic World Science & Technology Monitoring and Citation Institute (ISC), IRAN, ISLAMIC REPUBLIC OF

## Abstract

This study seeks best practices for when and how to apply short text topic modelling (STTM) techniques using natural language processing to semi-structured data collected as part of education research in order to provide accurate guidance for interventions and avoid misguided improvements in education practice. Student feedback was collected using short answer questions that resulted in 1,667, 1,592, and 1,376 expectations for faculty support, teaching assistant (TA) support, and peer support respectively as part of a larger survey conducted via convenience sampling in over 40 engineering courses offered at a single large university between 2016 and 2023. After cleaning and preprocessing the data, short text responses were analyzed using five unsupervised topic models implemented in Python: traditional models Latent Dirichlet Allocation (LDA), Latent Semantic Analysis (LSA), Non-Negative Matrix Factorization (NMF), and *k*-means and one deep learning model (BERTopic). Model performance was evaluated using topic coherence and exse than that expected by chance (internal performance metrics. Addressing a methodological gap in prior comparative studies that rely predominantly on machine-led evaluation, two approaches to establishing ground truth were evaluated: (a) keywords from each topic model guided manual (human) coding of the data (a machine-led approach); and (b) themes in the data were extracted and coded independently by a domain expert (a human-led approach). NMF achieved the highest average performance in two of the three datasets, reaching 75.6% accuracy, 75.7% F1-Score, and 0.63 interrater reliability for the peer support dataset and 72.6% accuracy, 72.0% F1-Score, and 0.57 interrater reliability for the TA support dataset. The human-led approach yielded higher accuracy and F1-scores for faculty and peer support but failed for TA support when the topics extracted by topic models did not align with themes identified by a domain expert. These findings highlight the need for humans to be involved in the analysis of short text data in contexts like education research where high performance is necessary to achieve appropriate rigor. Domain expert intervention also enables strategic use of topic models to optimize their use in qualitative data analysis.

## Introduction

Education research seeks to expand upon and address gaps in knowledge regarding how people learn and how practitioners can better facilitate learning. It aims to improve teaching practices and build inclusive learning environments that accommodate diverse student voices [1]. This requires both quantitative methods to address questions regarding what, how much, and how often students learn and teachers facilitate that learning, and qualitative methods to understand how and why they succeed or fail in their efforts. While quantitative methods remain more prevalent in peer-reviewed literature (particularly in STEM), qualitative research is gaining broader acceptance [2].

Among qualitative research methods, thematic analysis is acknowledged as one of the most accessible to a wide range of researchers [3,4]. Thematic analysis involves identifying patterns in datasets to extract relevant themes, offering a broader perspective than content analysis while preserving the richness of open-ended data. Unlike quantitative research with close-ended questions, thematic analysis can uncover previously unknown or emerging patterns, making it valuable across various fields and theoretical frameworks. However, thematic analysis faces several limitations. Its subjective nature makes it vulnerable to researcher bias, which requires multiple coders to assess reliability [5,6]. Small datasets also limit statistical analysis to support generalizability, while large datasets present challenges in terms of organizing themes coherently and providing the time-intensive human resources inherent to such analysis.

Artificial intelligence (AI), specifically natural language processing (NLP), offers potential solutions to these challenges. In particular, topic models are unsupervised NLP tools that explicitly seek similarities in the relationships between data points (i.e., words or sequences of words) [6], thereby making these models well suited for identifying patterns, an end goal that it shares with thematic analysis. A wide range of algorithms and approaches are available to capture these patterns and while a full review of topic models is outside the scope of this study, the comparative analysis presented herein examines four fundamentally different approaches to understanding and classifying language based on clustering, algebraic, probabilistic, and neural network techniques. Clustering offers a simple approach that groups similar uses of identical words together into individual clusters (i.e., topics), essentially treating words and words with little regard to context or the possibility that multiple topics may be present in a document. Probabilistic analysis groups documents with the same sets of words (i.e., word co-occurrences) together into topics but still fails to capture word (semantic) meaning even while considering some context of how words are used in combination with one another to express ideas. Matrix factorization (algebraic) methods seek to reduce superfluous information in text (i.e., noise) and actively seek out similarity instead of sameness among language patterns in documents, thereby offering additional insight into semantic meaning. And finally, more complex topic models based on neural networks offer the opportunity to capture the position of words in documents and their local and global (long-range) context by leveraging their pre-training on large and diverse datasets. Representing the straightforward to the complex, these four types of language models possess different benefits and drawbacks which must be explored and understood for specific types of datasets to develop actionable guidelines for their use. The goal of this comparative analysis is to develop such guidelines for use in the thematic (and related qualitative) analysis of student feedback and related data in education and education research.

### Topic models using clustering techniques

Traditional clustering approaches to topic modelling partition documents into groups based on similar word patterns without regard to what a word means (semantics) or the words which surround it (context). Among clustering approaches, *k*-means is computationally simple and the most widely used clustering technique in scientific and industrial applications including data mining [7]. *K*-means works by partitioning data into a pre-specified number of non-overlapping clusters by minimizing the distance between each document’s (numerical) vector representation and its corresponding cluster centroid. In addition to ignoring context and semantics, *k*-means is also limited by assigning each document to one and only one cluster and requiring all clusters to be spherical. Thus, poor performance can result when clusters vary significantly by size and density, when multiple topics overlap, or when variability exists in the initial partitioning of the data [8,9].

Despite these constraints, *k*-means is useful in short text topic modelling under specific conditions. For example, in a comparative analysis of inherently noisy and short Twitter and Reddit datasets, *k*-means proved to be the best performing clustering method in two of three datasets when documents were embedded into vectors using word2vec [8], a two-layer, neural network pretrained to learn word associations and semantics using large datasets [10,11]. In this same study, *k*-means also outperformed most clustering methods regardless of word embedding technique [8]. The conditions which enabled top level performance included incorporating considerations of word meaning (semantics) through the use of word2vec embedding, confining the data to a relatively narrow set of topics (discussions of Australian politics), and extracting ground truth from frequently occurring hashtags in documents rather than manual topic assignment [8]. Similar performance levels have been demonstrated for tweets associated with highly specific topics such as the World Cup soccer tournament, where *k*-means produced comparable results to the more advanced Non-Negative Matrix Factorization (NMF) approach after outlying points were removed to reduce noise [12]. Well-separated topics are also compatible with *k*-means clustering as illustrated by a study that analyzed health-related tweets and emails and found that *k*-means performed comparably to probabilistic models [9].

In addition to being useful in and of itself for capturing topics in data corpora under certain conditions, *k*-means can also serve as an effective baseline for topic modelling by establishing a minimum level of performance for comparison to more sophisticated techniques. Failure to perform at the level of *k*-means or beyond can suggest an issue with model parameter selection and optimization, a fundamental mismatch between the topic modelling approach and the underlying data corpus, unusual data characteristics that lend themselves to *k*-means, or a combination thereof. For these reasons as well as the appeal of simplicity and computational efficiency, *k*-means is included in this comparative analysis.

### Topic models based on probability

Like clustering methods, probabilistic topic models do not require any pre-existing information to identify hidden topics in texts. These models capture rudimentary global context by favoring word co-occurrences (i.e., the same words used together) across documents to identify topics. Of the probabilistic models available for topic modelling, Latent Dirichlet Allocation (LDA) is the most common [13] where the Dirichlet is a distribution of probability distributions chosen to model how topics are mixed in documents and how words are mixed in topics in such a way that the resulting mixtures are sparse and realistic. By leveraging the Dirichlet Distribution and evaluating which words and how often words co-occur in different documents, LDA determines how likely (probable) each possible topic is represented in each document, thereby allowing documents to exhibit multiple topics in varying proportions. While LDA ignores word order and local context which limits its capacity for capturing the meaning of words, it has nevertheless been widely used to sort and classify documents.

The negative impacts of LDA’s limited capacity for understanding semantics are minimized when analyzing data corpora containing longer texts with rich vocabulary overlap. Under these conditions, LDA has been able to successfully identify themes that align with human judgement and interest. For example, in the analysis of student feedback, Nanda et al. applied LDA to over 150,000 MOOC (massive open online course) learner comments averaging 16–17 words per document and found that topic assignments corresponded well with subsequent qualitative analysis, with the model successfully distinguishing themes related to course content, instructor quality, and learner satisfaction [14]. In another large study of over 110,000 student evaluations of teaching involving even longer texts, Sun and Yan found that LDA identified eight coherent topics; several of these topics correlated significantly with quantitative survey items, while others revealed themes that the survey questions did not address [15]. Similar alignment of themes with accepted principles of effective teaching practice have been demonstrated in the topic modelling of teacher self-assessment surveys, where LDA-identified topics mapped onto established frameworks for teaching practice [16]. In more applied research, LDA of learning behavior preferences among IT (information technology) students containing a rich vocabulary of 1,956 terms in 163 longer text documents not only revealed twelve distinct topics regarding what students expected of their teachers but clustering of the topics also grouped students according to their level of intellectual development. Doing so enables the development of practical recommendations for not only how instructors can meet student expectations but also how they can adjust teaching practice depending on where students are at [17]. These and related studies not only demonstrate the broad applicability of LDA to different types of data but also suggest that LDA performs particularly well when three conditions are met: documents contain sufficient words to establish co-occurrence patterns, vocabulary overlaps substantially across documents addressing similar themes, and topics are reasonably distinct rather than highly correlated.

Unfortunately, when texts are short, LDA can struggle. Short texts provide limited word co-occurrence information which leads to sparse document representations and unstable topic assignments. This unstable behavior has been consistent demonstrated across a wide range of short text datasets including tweets, news tidbits, snippets from web-based searches, and texts extracted from question and answer forums [18]. Adding to this instability, the assumption that word order does not matter becomes problematic when word order is meaningful. LDA also assumes that topics are independent with little overlap; this is particularly problematic when analyzing student or teacher experience in education, because what goes on in the relatively small world of the classroom leads to themes or topics that inherently overlap or co-occur. Additionally, LDA’s output can be sensitive to hyperparameter settings and initialization, which introduces variability across runs that complicates replication. To address some of these limitations, researchers have developed LDA variants like Correlated Topic Models which relax the independence assumption, Biterm Topic Models that aggregate word pairs to address sparsity, and seeded or guided approaches which incorporate domain knowledge through predefined word lists [19]. These extensions improve performance in specific contexts but add complexity and do not fully resolve the underlying dependence on word co-occurrence. The bottom line is that when documents contain same word patterns that are meaningful to the goal of a particular topic modelling effort, LDA can be powerful, accurate, and efficient. Its inclusion in this comparative analysis allows direct evaluation of whether the probabilistic framework offers advantages over clustering for student feedback data, and whether its known limitations with short text are offset by its capacity to model mixed-topic documents.

### Topic models using matrix factorization

Matrix factorization breaks down a single matrix into multiple smaller ones which contain hidden (latent) information about features of the original matrix. Two of the most common matrix factorization methods used for topic modelling are Non-Negative Matrix Factorization (NMF) and Latent Semantic Analysis (LSA). LSA uses singular value decomposition (SVD) to break the original matrix that represents a document corpus down into three matrices while NMF iteratively adjusts two matrices to minimize the difference between their product and the original matrix. Both methods produce a document-by-topic and a topic-by-word matrix which can be analyzed to identify similarities between documents and similarities between words respectively. The capacity to identify such similarities enables both methods to detect synonyms and integrate semantic meaning into the topic modelling process. The key difference between the two methods is that LSA allows for negative values in the component matrices which can capture more complex and nuanced language usage while NMF does not allow for such negative values, thereby making the resulting matrices easier to understand and interpret [20,21].

In education, LSA has been particularly useful in evaluation (i.e., grading) of student work because it is adept at mimicking human judgements of similarities between texts. For example, LaVoie et al. [22] demonstrated a correlation of 0.94 between LSA-driven automated scoring and human scoring of responses from consequence tests which asked students to assess the outcomes of a decision or action. Similar levels of agreement between LSA and human judgement have been demonstrated for language proficiency tests [23], questions which require analytical reasoning of psychology lectures [24], and prompts requiring students to summarize technical texts [25]. In some tests such as those measuring creativity, LSA has even performed better at modelling the meaning and originality of responses than traditional human assessments [26]. Due to its alignment with human judgement, LSA has also been integrated into successful, commercially available products for automated essay scoring [27,28]. Perhaps more in line with thematic analysis, however, LSA has also been shown to capture key ideas in student feedback. For instance, in a medical school course where students submitted anonymous reflections on gender differences in medicine, LSA was able to identify ten topics that captured over half of the variance in the data [29]. Reasonable topic coherence has also been demonstrated when using LSA to analyze student feedback on teaching quality in multiple courses over an academic year [30].

While LSA is able to capture more complex relationships between texts and thereby produce results that often coincide with human raters and human judgement, non-negative matrix factorization (NMF) offers the distinct potential for providing improved topic coherence (i.e., words within a topic frequently appear together in documents) and interpretability (i.e., words representing a topic are meaningful to human judgement) [31]. Greater coherence and interpretability, in turn, can make it easier to connect the output of NMF to themes that are both formulated by and relevant to human endeavors. The use of NMF for analyzing student work, student feedback, and other textual data from education and education research has been very limited compared to studies involving LSA. However, a broad range of studies have demonstrated superior topic coherence with NMF-based topic models outside of education. Compared to LDA, NMF topic models show consistently better statistical measures of topic coherence for analyzing data corpora extracted from Google snippets [32], news headlines [32,33], news descriptions [32], on-line question and answer forums [32], and restaurant reviews [34]. These benefits of NMF are not limited to statistical coherence measures but extend to both human and large language model measures of coherence as well [33]. Further, NMF has been shown to outperform LDA by producing more distinct topics with lower overlap in the analysis of news articles and Wikipedia pages, suggesting that NMF is better suited to niche or non-mainstream data [35] which are common in education and other specialized domains. As evidence of this, one of the few studies exploring the use of NMF in education showed, in examining student feedback regarding mental health and remote learning during the COVID-19 pandemic, that topics discovered by NMF were similarly coherent and more numerous and granular than those found by LSA and LDA [36]. This improvement in granularity has been duplicated in studies comparing *k*-means clustering with NMF modelling of World Cup tweets [37] and also of aviation accident reports, although with a corresponding decline in topic coherence compared to LDA [38]. Noisy data such as that obtained from tweets in real time also appears to compromise the ability of NMF to produce interpretable topics compared to other topic modelling techniques [39]. Thus, while the potential of NMF to produce more coherent, more distinct, and more interpretable topics has been clearly demonstrated in multiple previous studies, these benefits are not guaranteed and actual performance is highly dependent on the characteristics of the underlying data.

While matrix factorization techniques have proven and distinct advantages over LDA and traditional clustering techniques, they still rely largely on mathematical transformations rather than contextually rich language models. This limitation brings us to the next evolution in topic modelling: neural network approaches which rely on pretrained large language models (LLMs).

### Topic models based on neural networks

Topic models based on neural networks differ fundamentally from traditional approaches to topic modelling in how words are embedded into numerical representations. *k*-means, LDA, LSA, and NMF typically use bag-of-words embedding which convert words to numbers based on how often they occur in a document (i.e., frequency-embedding) or add a layer of computation to de-emphasize common words while highlighting rare words (e.g., TF-IDF -- term-frequency, inverse-document frequency). These methods lack consideration for the order of words, long-range global context in a document, or other clues regarding semantics (meaning). This distinction matters because these methods often fail to recognize such semantic similarities as those between “helpful” and “supportive” if they never appear together in the dataset or are used in different language patterns. Sophisticated neural networks can overcome these barriers to capturing the meaning of words by considering how and where words are used in text relative to other words. Of these neural approaches to word embedding, BERT (Bidirectional Encoder Representations from Transformers) is widely used and represents words based on their surrounding context rather than fixed dictionary definitions [40]. BERTopic then integrates BERT into a topic modelling pipeline where the position of BERT-embedded words is encoded, the dimensions of the embedded matrix are reduced, the reduced data are clustered together into topics, and representative terms (e.g., words) for each topic are strategically extracted [41].

BERTopic performs well when documents express similar ideas with varied vocabulary. For example, in the analysis of MOOC student discussion forums, Khodeir and Elghannam [42] found that BERTopic outperformed both LDA and LSA approaches and was competitive with NMF in terms of both statistical topic coherence and topic diversity scores because it captured semantic content that co-occurrence-based models missed. This advantage reflects BERTopic’s ability to recognize that phrases like “need help immediately” and “struggling with deadline” convey similar urgency despite sharing no words. BERTopic modelling in other domains has demonstrated similar performance benefits. Superior statistical topic coherence of BERTopic has been demonstrated in the analysis of COVID-19 vaccination tweets using multilingual embedding models [43], adolescent health tweets [44], disaster-related tweets [45] and news headlines [46], but when topic coherence was evaluated using human or large language model evaluation, BERTopic consistently outperformed not only LDA but also NMF [47]. And, while BERTopic and NMF often perform similar to one another, BERTopic initially produces more topics which can offer unique insight into data that traditional methods cannot [12]. BERTopic can also complement NMF by providing finer granularity for certain topics while NMF obtains comparable granularity in other topics in the same dataset [36]. BERTopic, however, does not always perform better than traditional topic models. For instance, LDA has been shown to outperform BERTopic when comparing topic diversity from topic models in multiple studies [43].

BERTopic performance can suffer when considering texts in different languages or in specific domains because pretrained embeddings may not represent specialized terminology well. For instance, educational feedback often contains domain-specific terms such as “office hours” and “curve the exam” that general-purpose embeddings may miss. BERTopic also inherits the single cluster limitation of *k*-means where each document is assigned to exactly one topic, which can misrepresent texts that address multiple topics or belong to different themes. Further, generating embeddings can also be computationally expensive for large datasets, though this cost is incurred once and can be reduced through caching. Thus, when traditional topic models work and work well enough, the additional computational complexity introduced by BERTopic is not warranted. Superior performance by BERTopic, however, can not only be desirable for the task at hand but can also offer insight into the characteristics of the data that make it unsuitable for traditional models and in turn vulnerable to errors using any topic model.

### Previous comparative analyses of topic models

This study focuses on comparing different approaches to topic modelling of short texts in the education domain. In this spirit, our comparative analysis investigates baseline models associated with four different approaches (clustering, probabilistic, matrix factorization, and neural networks) rather than variants or modifications of these baseline approaches with the goal of developing broad guidelines for how researchers and practitioners in education can strategically use NLP in their work. Each approach has different advantages and disadvantages which makes no single choice the right choice for every dataset. Previous comparative analyses have underscored that there is no one-size-fits-all topic model for short texts (Table 1). Considering 16 comparative analyses conducted between 2019 and 2025, five analyses (31.3%), four analyses (25%), and seven analyses (43.8%) identified NMF, LDA, and neural network-based models (including BERTopic) respectively to be among the top performers. One study also found that LSA performed best, outperforming LDA and several variants of LDA.

**Table 1:** Comparative analyses of short text topic models (STTMs).

*Ref*	*Algorithms*	*Dataset*	*Average Words or Tokens*	*Size of Corpus*	*Performance Measures*	*Top Performers*
Chen et al., 2019 [32]	BTM (standard), DMM (two variants) LDA (standard), LDA (two variants), NMF (standard), NMF (one variant)	Google Snippets	14.3	12,284	Topic Coherence^1^	NMF
		News	11.8	35,592		
		Stack Overflow	7.25	19,783		
		XinlangNews	4.38	8,966		
		TMNtitles	3.46	29,487		
		Tweets (general)	5.81	102k		
Qiang et al., 2022 [18]	BTM (standard) DMM (four variants) LDA (standard) oProb (one variant) SA (two variants)	Pascal Flickr	5.37	4834	Classification^2^ Topic Coherence^1^	DMM
		Stack Overflow	5.03	20K		
		Biomedicine	7.44	19448		
		Tweets (Queries)	8.55	2472		
		Google News	6.23	11,109		
		Search Snippets	14.4	12,295		
Albalawi et al., 2020 [47]	LDA (standard) LSA (standard) NMF (standard) DimRed (two variants)	Newsgroup data	28	20K	Topic Coherence^1^, Classification^2^	LDA
Doan & Hoang, 2021 [48]	LDA (standard) LDA (two variants) NMF (standard) NN (five variants)	20News	73.52	15,465	Perplexity^3^, Classification^2^	NN
		W2E-title	6.9	105,522		
		Web Snippets	14.42	12,295	Topic Coherence^1^	NN
Lossio-Ventura et al., 2021 [9]	BTM (standard) DMM (one variant) LDA (standard) LDA (three variants) LSA (standard) *k-*means	Tweets (Health)	~9	286,971	Internal Cluster Quality^4^	LDA OProb
		Emails (Health)	31.78	50,000	External Cluster Quality^5^	LSA *k*-means
Egger & Yu, 2022 [12]	LDA (standard) NMF (standard)	Tweets (COVID Travel)	NR^6^	31,800	Qualitative Comparison	NMF
	LLM (one variant) NN (one variant)					LLM
Murshed et al., 2020 [49]	BTM (standard), LDA (standard) LDA (one variant) NMF (standard) oMF (one variant) oProb (three variants SA (two variants)	Tweets (COVID)	NR^6^	42k	Topic Coherence^1^ External Cluster Quality^5^ Classification^2^	oProb
		Tweets (bullying)	NR^6^	20K		
Goyal et al.,2023 [50]	LDA (two variants) NMF (standard)	Image Captions	9.7	10K	Classification^2^ Topic Coherence^1^	NMF
					Internal Cluster Quality^4^	LDA
					External Cluster Quality^5^	LDA
Muthusami et al., 2024 [51]	LDA (standard) NMF (standard)	Tweets (stance detection)	NR^6^	2914	Topic Coherence^1^ Internal Cluster Quality^4^	NMF
Doi et al., 2024 [52]	LDA (two variants) NN (four variants)	Tweets	5.47	2k	Topic Coherence^1^, Topic Diversity^6^	NN
		GoogleNews	5.25	11k		
		StackOverFlow	4.71	19,000		
Hayat et al., 2024 [36]	NN (one variant) LDA (standard), LSA (standard) NMF (standard)	Student feedback (well-being)	NR^7^	375	Topic Coherence^1^, Qualitative Comparison	NN
		Student feedback (remote learning)	NR^7^	321		
Sheils et al., 2024 [53]	LDA (standard), NMF (standard) NN (two variants)	RateMyProfessors	NR^7^	1M	Topic Coherence^1^, Internal Cluster Quality^4^	NN
Barker et al., 2024 [19]	LDA (standard) oProb (two variants)	Discussion Forum (Statistics Ed)	NR^7^	946	Qualitative Comparison	oProb
Medvecki et al., 2024 [43]	LDA (standard), NMF (standard) NN (three variants)	Tweets	NR^7^	3,286	Topic Coherence^1^	NN
					Topic Diversity^6^	LDA
Babaloa et al., 2024 [33]	LDA NMF NN	News Headlines	6.45	149,679	Topic Coherence^1^	NMF NN
Feng et al., 2025 [44]	LDA NMF NN (three variants)	Health Tweets	NR^7^	64,441	Topic Coherence^1^ Topic Diversity^6^	NN

^1^A measure of the degree to which words belong to a topic semantically.

^2^A measure of how well topics support predicting document labels (Accuracy and F1-score).

^3^A measure of how well a model predicts unseen text data.

^4^A measure of topic quality based on model structure.

^5^A measure of how well topic clusters align with known categories.

^6^A measure of range and distinctiveness of topics.

^7^Not reported for this dataset, but Tweets are typically limited to 280 characters (46–55 words).

BTM (Biterm); DMM (Dirichlet Multinomial Mixture) LDA (Latent Dirichlet Allocation);

oProb (Other Probabilistic topic models); oMF (Other Matrix Factorization topic models);

SA (Self Aggregating topic models); LSA (Latent Semantic Analysis);

DimRed (Non-LSA Dimension Reduction models);

NN (neural network based topic models including BERTopic).

While the comparative analyses outlined in Table 1 add further justification for including LDA, NMF, LSA, and BERTopic in this study, several topic models that performed well in previous comparative analyses were rejected for consideration. Dirichlet Multinomial Models (DMMs) and Biterm topic models (BTM) were not considered primarily because they are designed for sparsity and optimized for ultra-short texts (15 words or less) which were shorter than most documents in the datasets used in this analysis. Self-aggregating (SA) models were also rejected because they merge shorter texts into longer documents prior to topic modelling. Doing so with research data would run the risk of blurring and compromising distinct opinions. Furthermore, NMF variants such as Semantic-assisted NMF (SeaNMF) and Knowledge-guided NMF (KGNMF) were not considered because they are specifically designed to handle word co-occurrence sparsity. This type of sparsity is often characteristic of data with a wide range of language use, such as that found in the public domain, including social media. Given that the data collected for this study was guided by a specific prompt and therefore was much more focused than public domain datasets, these techniques were deemed less appropriate and not considered in this analysis.

### This Study

While the comparative analyses summarized in Table 1 demonstrate that different models excel under different conditions, they offer limited guidance for researchers working outside the social media and news domains that dominate this literature. Education researchers analyzing student feedback face this gap directly: which model should they choose, and what happens if they choose wrong? Misclassified student responses can lead to flawed interpretations of what students need and want from their instructors, teaching assistants, and peers. Themes that matter may go undetected while false patterns are mistaken for meaningful insights. These errors can misdirect efforts to improve teaching and waste resources on interventions that address the wrong problems.

Beyond this general lack of guidance, several limitations prevent findings from prior comparative analyses from translating directly to education research and practice. First, most previous studies rely on social media data (tweets, Reddit posts) or news corpora that differ substantially from the semi-structured feedback commonly collected in educational settings. Chen et al. [32], for example, compared multiple variants of BTM, DMM, LDA, and NMF across six corpora of tweets, news articles, and Stack Overflow posts, and Babalola et al. [33] compared LDA, NMF, and BERTopic on a corpus of news headlines; like most studies in Table 1, neither considered education-related text. Student responses to prompts about instructional support use domain-specific language and exhibit different characteristics than public discourse, making it unclear whether approaches for analyzing Twitter or news headline data generalize to analyzing texts related to education. One of the few prior studies of student feedback, by Hayat et al. [36], evaluated four STTMs and demonstrated that no one model fits all feedback; NMF provided the best results for one dataset while BERT provided best results for the second dataset in the study, highlighting the need for best practices for matching a topic model to the characteristics of a particular dataset or stage in analysis of that dataset. Furthermore, most prior comparative studies evaluate models using statistical coherence measures, often supplemented by automated assessments, without examining whether the resulting topics are interpretable or actionable for practitioners who must act on the findings [32,33].

By evaluating both statistical metrics and the practical interpretability of topics for educators, this study bridges the gap between computational performance and pedagogical relevance. These considerations lead to our first research question:

*(RQ1) How do topic models based on natural language processing (NLP) perform when applied to datasets used for education research and practice?*

Finally and most critically, nearly all prior comparative analyses of STTMs, including Hayat et al.’s study of student feedback [36], evaluate topic models using a single approach to ground truth, typically one where human coders label data after seeing model output (a machine-led approach) [12,19] or where ground truth is derived from metadata such as hashtags rather than independent human judgment [8]. This methodological choice may inflate apparent model performance by biasing evaluation toward the patterns the model has already identified. The alternative, having domain experts identify themes independently without seeing model output (a human-led approach), remains largely unexplored in comparative studies, despite its potential to yield different conclusions about which model performs best.

This study addresses this methodological blind spot by systematically comparing machine-led and human-led approaches to establishing ground truth via the following additional research question:

*(RQ2) Are there significant differences in machine-led vs. human-led application of STTMs that indicate bias in the former approach?*

By comparing five topic models (k-means, LDA, LSA, NMF, and BERTopic) across three student feedback datasets (faculty, TA, and peer support) collected in engineering courses, this analysis provides evidence specific to the education domain and exceeds the scope of the only prior study on student feedback [36].

## Materials and methods

This comparative analysis was conducted as part of a larger study investigating the relationship between instructional support and student engagement within a single institution across multiple academic years [54]. A survey was used to examine these relationships and included demographic, Likert-scale, and short answer questions. Short text responses from students to the following three short answer questions were extracted from the survey to support this study:

- Faculty Support: *“What one action can your professors at <institution name> take to best support you in your classes (please be as specific as possible)?”*
- TA Support: *“What one action can your TAs at <institution name> take to best support you in your classes (please be as specific as possible)?”*
- Peer Support: *“What one action can students in your <coursename> class take to improve your educational experience (please be as specific as possible)?”*

A diverse population of students responded to the survey. A majority were male (74.3%), White (37.2%), Asian American (44.1%), or domestic (84.6%), but significant numbers of Black, mixed race, Latino/a, international, and female students also responded to the survey. Student respondents also spanned 43 engineering courses across three different time periods corresponding to traditional learning before (32.2%) and after (21.6%) the COVID-19 pandemic and remote learning during the pandemic (46.2%). Descriptive statistics for word and character counts for each group of student responses (i.e., datasets) before and after data preprocessing are summarized in Table 2.

**Table 2:** Dataset characteristics.

	Word Count							Character Count					
Data	Raw Data				After Preprocessing*			Raw Data			After Preprocessing*		
	*N*	*M*	*Mdn*	*SD*	*M*	*Mdn*	*SD*	*M*	*Mdn*	*SD*	*M*	*Mdn*	*SD*
Faculty	1,676	23.6	17	22.0	10.1	8	8.3	140	103	125	72.9	57	60.7
TA	1,592	19.9	14	18.9	8.3	6	6.9	115	84	104	58.8	46	49.4
Peer	1,375	16.2	11	16.1	6.4	5	5.7	94.6	67	92.6	45.5	33	43.1

*N* (Number of Documents), *M* (Mean), *Mdn* (Median), *SD* (Standard Deviation).

*Data preprocessing included converting text to lowercase, expanding contractions, removing punctuation, special characters, and numbers, POS (part of speech)-aware lemmatization using spaCy, and removing stopwords (default English + custom domain-specific terms).

### Procedures

IRB (Internal Review Board) approval (STUDY00000378) was obtained to recruit and survey undergraduate students using convenience sampling from 43 engineering classes, beginning on October 26, 2016. While the exempt status under which this study was approved did not require continuing review, data collection was discontinued on June 15, 2023. Instructors were asked to offer the survey to their students within two to three weeks of the end of the term in which the course was offered. Instructors offered an incentive to students to complete the survey, with a nominal amount of extra credit being the most popular choice; extra credit has been shown to be a highly effective motivator for college students [27]. For all but one class in the pre-COVID and ERT (emergency remote teaching during the COVID-19 pandemic) time periods, the survey was hosted by an institution-specific survey tool (Catalyst WebQ) and students accessed and completed the survey via a link in the learning management system for the course (Canvas) within one to three weeks of the instructors publishing the survey. In the remaining course (a 2016 pre-COVID offering), students completed a paper version of the survey in class. In the post-COVID period, student responses were collected using either Catalyst WebQ (2022) or Google Forms (2023). Instructors were not provided with any survey responses but instead were provided with a list from the researchers of names and percentage of questions completed by each student so that grades could be adjusted according to the incentive offered to students. All participation was voluntary, and students were offered extra credit regardless of whether they granted consent for their responses to be used in the research because institutional IRB required that those students who did not provide consent not be excluded from taking the survey. Participants provided informed consent either in written form (for the paper form of the survey in the 2016 pre-COVID course) or in electronic form via a unique link to their student network ID. No minors participated in the survey and less than 5% of those who completed the survey did not offer consent and were eliminated from the dataset.

### Data analysis

Five topic models were compared for each of the three instructional support datasets. A more thorough explanation of the algorithms and processes underlying each model can be found for *k*-means analysis in [55]; for LDA in [13]; for LSA in [20]; for NMF in [31], and for BERTopic in [41]. Each of the five models provides a unique approach to uncovering meaningful topics from textual data which can subsequently be used to define themes that describe that data. To facilitate a one-for-one comparison among models, default parameters were used for all five models and *k*-means clustering rather than hierarchical clustering was used in the BERTopic model to ensure that all five models conformed to the same number of topics.

**Fig 1:**
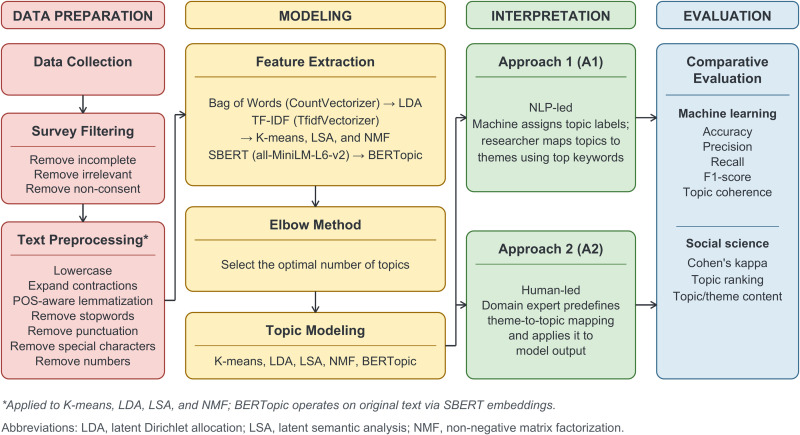
Data analysis process flow.

Using these five models, all three datasets were analyzed using the process shown schematically in Fig 1. The survey data first underwent a comprehensive cleaning process to correct spelling errors, remove identifying information, and delete responses from students who did not provide consent. The data were then filtered to remove responses that were blank or did not provide any suggestions for improved support. Next, all documents in the dataset were preprocessed using the sklearn library [56] in Python 3 to reduce noise by converting all text to lowercase, expanding contractions, removing punctuation, special characters, and numbers, performing POS-aware lemmatization using spaCy, and removing stopwords (default English and custom domain-specific terms). After preprocessing, the data were embedded (converted to numerical/vector form). For the LDA, LSA, NMF, and *k*-means models, word embedding was performed using the best performing of (a) a basic bag of words (BOW) approach that tracked the frequency each word in each document or (b) the more advanced term frequency-inverse document frequency approach (TF-IDF) [56].

After word embedding, all topic models were initially analyzed for between three and seven topics. The optimal number of topics was determined using the elbow method [57], which involves plotting the number of topics against the explained variance associated with the number of topics and identifying the point where the increase in explained variance starts to diminish with increasing numbers of topics, forming an “elbow” in the plot [58]. Once the optimal number of topics was determined, the performance of each topic model (*k*-means, LDA, LSA, NMF, BERTopic) was evaluated using topic coherence as an internal performance metrics as well as external performance metrics which were evaluated against ground truth in two different ways:

- Approach 1 (A1): The top ten words associated with each topic in each model were distilled down to the most relevant keywords by a domain expert. This information was then used by another domain researcher to code the dataset. This approach essentially evaluated how well *machine-led* coding worked for modelling the data. The resulting topics may or may not correspond to underlying themes.
- Approach 2 (A2): When provided with the optimal number of topics in each dataset, a domain expert independently identified themes in the data corresponding to this optimal number without referencing model-generated information. Once these themes were identified, the domain expert then coded the data. In this way, this approach assessed how well machine-generated topics replicated *human-led* theme assignment

Four sets of performance metrics were computed for each of the 30 models evaluated in this analysis. Internal performance (i.e., that which does not require a ground truth or external assessment) was evaluated using topic coherence. Topic coherence measures how semantically similar the words belonging to a particular topic are to one another [59]. Higher topic coherence implies greater interpretability (i.e., topics that make sense to human interpretation) and in the case of thematic analysis, a greater likelihood that the topics correspond to meaningful themes. In this study, topic coherence was calculated using *U*_Mass_ which measures how often two of the top words associated with a topic appear together in documents within a corpus and averages these pairwise coherence scores to arrive at a global coherence score. The second set of performance metrics used in this analysis emphasized traditional external performance metrics used in the assessment of machine learning models [60–62] based on true positives (TP), true negatives (TN), false positives (FP), and false negatives (FN):

- Accuracy: The ratio of (TP + TN) to (TP + TN + FP + FN)
- Cohen’s Kappa (κ): While not traditionally used to characterize performance of a topic model compared to ground truth, κ serves as an additional measure of accuracy by evaluating the agreement between topic model results and codes assigned to documents by manual (human) analysis while accounting for agreement occurring by chance [63].
- Precision: The ratio of TP to (TP + FP)
- Recall (aka sensitivity): The ratio of TP to (TP + FN)
- F1-score: The harmonic mean of precision and recall [64]. The average (macro) and weighted average (sample weighted) F1-scores were calculated using the individual F1-scores for each topic in each model while the aggregate (micro) F1 score which uses the total number of FP, FN, and TP over all topics was also calculated.

While these initial two sets of performance metrics were quantitative in nature, an additional metric allowed for qualitative assessment of performance among the models. The nature (or quality) of themes was subjectively assessed using the top keywords associated with a topic as determined by the STTM models. These word-based (qualitative) topic and theme descriptions were then compared among models and between topic models and human theme assignment to assess how well the topic models replicated human judgement. Further, two additional performance metrics were used to compare model performance emphasizing an aggregate perspective on the data by comparing the *number of themes* identified by the topic models vs. domain expert (human) assessment and the *ranking of themes* in terms of frequency.

While not directly evaluating performance, two additional measures were evaluated by a domain expert in order to facilitate explanation and discussion of results of the topic models: (a) ambiguity, which coded each response as distinctly associated with a single topic or theme (non-ambiguous) or vulnerable to confusion with one topic over another; and (b) multiple topics, indicating that a student response (document) could be reasonably assigned to more than one topic.

## Results

For the three datasets, an elbow plot considering the variance embodied by the topics indicated that three topics was an optimal choice for all five topic models. Three topics that were closely aligned with pedagogical themes were subsequently sought out via an independent analysis by a domain expert. Some themes were similar across datasets and some were unique to a single dataset, resulting in a total of five distinct themes that represented the data:

- Theme #1, *interactions* was present in all datasets and represented students’ desire for interactions with others to enhance learning. The specific nature of interactions (e.g., with peers or instructors) and formats (e.g., office hours, forums) varied among the three datasets but shared a consistent underlying focus on the importance of engaging with others to student learning.
- Theme #2, *teaching practice* focused on students’ desire for TAs and faculty to be more effective in their teaching practice, whether through better delivery in the classroom, providing additional resources to support learning, synchronizing faculty-led lectures with TA-led quiz/recitation and lab sections, or similar adjustments. This theme excluded practices that emphasized interactions among students and instructors.
- Theme #3, *examples and experience* called for faculty and TAs to provide ample opportunities to reinforce teaching practice with experiential learning opportunities including appropriate homework, exams, practice problems, scaffolded examples, active learning/problem solving experiences, and laboratory support.
- Theme #4, *questioning* uniquely applied to the peer support dataset. Students desired that their peers not only ask more questions but ask more appropriate questions in class to support a more effective and interactive atmosphere for their learning.
- Theme #5, *civility* also applied only to the peer support dataset and included a broad range of civil behaviors expected of peers in class including but not limited to refraining from disruptive talking and other distracting activities as well as being on time to class.

These five themes were used by two independent raters with domain expertise to code a subset of 100 responses from each of the three datasets to evaluate interrater reliability (Table 3). In the case of faculty support, low κ scores triggered a second pass at the data after code/theme refinement to achieve sufficient reliability in the traditional (manual) coding process.

**Table 3:** Interrater reliability (Approach 1 and 2).

	Cohen’s Kappa (κ)		
	Faculty Support	TA Support	Peer Support
Approach 1	0.64/0.77*	0.71	0.73
Approach 2	0.74	0.72	0.75

*A second pass was conducted after revising/clarifying themes.

Once codes were refined and sufficient reliability established, the primary domain expert coded the remaining data without any further access to the topic model results (Approach 2). For Approach 1, another domain expert used the most relevant and informative of the top ten words (i.e., keywords) associated with each topic identified by the NLP topic models (Table 4) to guide manual coding of the data. These codes, whether they aligned with pedagogically relevant themes or not, became the ground truth for the purposes of computing many of the performance metrics.

**Table 4:** Keywords used to manually code data using Approach 1.

	Topic	Relevant Keywords
Faculty Support	0	*k*-means (lecture, material, note, record, clear, recording); LDA (lecture, record, online, flexible); LSA (lecture, record, recording, note, homework); NMF (lecture, record, note, slide); BERTopic (lecture, note, slide, recording)
	1	*k*-means (example, problem, practice, exam, homework); LDA (problem, example, material, practice, homework); LSA (example, problem, practice, exam); NMF (problem, example, exam, homework, solution); BERTopic (problem, practice, exam, example, homework)
	2	*k*-means (office, hour, offer, available, email); LDA (hour, office, question, ask, available, help); LSA (office, hour, available, answer); NMF (hour, office, offer, help, available, email); BERTopic (office, hour, help, available, online)
TA Support	0	*k*-means (lab, problem, section, example, material); LDA (lab, problem, section, example, material, homework); LSA (lab, problem, example, quiz); NMF (lab, problem, example, material, practice); BERTopic (problem, material, example)
	1	*k*-means (office, hour, offer, available, help); LDA (hour, office, help, offer, email); LSA (office, hour, help, available, answer); NMF (office, hour, offer, available, help); BERTopic (hour, office, email, time, help, available)
	2	*k*-means (question, answer, ask, available, help, email); LDA (question, answer, ask, help, available); LSA (question, answer, problem, help); NMF (question, answer, available, ask, email, help); BERTopic (lab, help, time, question)
Peer Support	0	*k*-means (help, group, participate, respectful, study, discussion); LDA (group, study, discussion, breakout); LSA (study, group, help, talk); NMF (study, group, help, willing, discussion); BERTopic (talk, group, help, study, discussion)
	1	*k*-means (talk, distract); LDA (professor, talk, participate, respectful, distract); LSA (talk, participate, distract, respectful); NMF (talk, participate, distract, respectful, quiet); BERTopic (ask, think, understand)
	2	*k*-means (question, ask, answer, think); LDA (help, willing, open, understand); LSA (question, ask, answer, participate); NMF (ask, question, answer, think, afraid); BERTopic (question, ask, answer, insightful, valuable)

### Quantitative measures of topic model performance

#### Faculty Support Dataset.

Quantitative, internal and external performance metrics for the topic modelling of student responses to prompts regarding faculty support are summarized in Table 5. Across all metrics, Approach 2 (A2) to determining ground truth performed consistently better than Approach 1 (A1). In a one-for-one comparison of topic models within approach A2, *k*-means topic models produced the highest external performance scores followed by NMF models while BERTopic produced the most consistent performance (as measured by the standard deviation of performance across the three topics). In terms of internal performance (measured by *U*_mass_ topic coherence), NMF produced both the best coherence scores and the smallest variation in coherence across the three topics.

**Table 5:** Performance metrics for faculty support topic models. (A1: Ground truth Approach 1; A2: Ground Truth Approach 2; *U*_mass_: Topic Coherence).

		*All Metrics are Percentage (%)*									
Theme	Metric	*k*-means		LDA		LSA		NMF		BERTopic	
		A1	A2	A1	A2	A1	A2	A1	A2	A1	A2
Examples and Experience	Accuracy	82.0	92.3	59.4	65.6	70.8	69.8	65.5	67.8	66.4	67.8
	Cohen’s κ	0.37	0.74	0.19	0.32	−0.16	−0.16	0.30	0.35	0.31	0.34
	Precision	48.8	82.2	27.2	34.5	0.50	1.00	32.8	36.3	33.3	36.0
	Recall	47.5	75.2	77.6	92.7	0.30	0.60	91.9	95.5	90.5	92.0
	F1-Score	48.1	78.5	40.2	50.3	0.40	0.80	48.4	52.6	48.7	51.7
	*U*_mass_	−2.74	−2.74	−4.46	−4.46	−2.07	−2.07	−2.21	−2.21	−10.6	−10.6
Interactions	Accuracy	77.8	81.8	77.0	80.6	71.0	74.8	79.9	83.5	79.2	78.9
	Cohen’s κ	0.41	0.47	0.47	**0.53**	0.16	0.18	0.48	**0.55**	0.49	0.46
	Precision	91.4	91.8	66.7	66.3	100.0	100.0	86.2	85.6	76.4	67.6
	Recall	36.4	41.3	60.4	67.9	12.1	13.7	46.5	52.2	53.4	53.4
	F1-Score	52.0	57.0	63.4	67.1	21.6	24.1	60.4	64.8	62.8	59.7
	*U*_mass_	−2.13	−2.13	−2.09	−2.09	−2.47	−2.47	−2.73	−2.73	−1.75	−1.75
Teaching Practice	Accuracy	61.4	75.5	52.2	57.2	43.2	46.7	67.5	67.0	65.4	61.9
	Cohen’s κ	0.23	0.50	0.04	0.16	−0.13	−0.10	0.35	0.35	0.30	0.25
	Precision	57.7	69.8	54.1	73.4	45.6	49.3	75.5	79.0	75.4	74.1
	Recall	81.5	93.5	21.6	27.8	77.9	79.7	50.4	49.9	44.4	41.3
	F1-Score	67.6	79.9	30.9	40.4	57.5	60.9	60.5	61.2	55.9	53.1
	*U*_mass_	−2.45	−2.45	−2.13	−2.13	−2.89	−2.89	−2.26	−2.26	−2.25	−2.25
*Overall Performance*											
Average Accuracy		60.6	74.8	44.3	51.7	42.5	45.6	56.4	59.1	55.5	54.4
Average Cohen’s κ		0.32	0.56	0.22	0.32	−0.05	−0.03	0.37	0.40	0.36	0.34
Average *U*_mass_		−2.44	−2.44	−2.89	−2.89	−2.48	−2.48	−2.40	−2.40	−4.88	−4.88
Macro F1		55.9	71.8	44.8	52.6	26.5	28.6	56.4	59.5	55.8	54.8
Micro F1		60.6	74.8	44.3	51.7	42.5	45.6	56.4	59.1	55.5	54.4
Sample Weighted F1		59.0	73.0	43.3	50.0	35.6	38.9	58.3	60.6	56.9	54.8

#### TA support dataset.

Quantitative, internal and external performance metrics for the topic modelling of student responses to prompts regarding TA support are summarized in Tables 6 and 7 for Approach 1 and Approach 2 respectively. The three topics determined by the topic models did not align with the three themes identified independently by a domain expert (Approach 2). Instead, the topic models generated topics associated with *availability*, question and answer opportunities (*Q&A*), and *teaching practice* while the domain expert identified themes of *examples and experience*, *interactions*, and *teaching practice* (similar to the themes identified in the faculty support dataset). The *availability* and *Q&A* topics were, in combination, aligned with the *interactions* theme, while the *examples and experience* theme was absorbed into the *teaching practice* topic identified by all five topic models. This misalignment between topics and themes restricted the performance metrics to two topics (Table 7) rather than the three topics enabled by Approach 1 (Table 6). Because Approach 2 was fundamentally unable to align themes with topics, it was considered the less ideal of the two approaches. In a one-for-one comparison of topic models using Approach 1, NMF topic models produced the highest average external performance scores (accuracy, F1-score) while LSA produced the best per-topic performance scores followed by NMF models. LDA produced the most consistent performance (as measured by the standard deviation of performance across the three topics) followed closely by NMF. In terms of internal performance (measured by *U*_mass_ topic coherence), *k*-means produced both the best average and per-topic coherence scores as well as the smallest variation in coherence across the three topics.

**Table 6:** Performance metrics for TA support topic models (Approach 1). (*U*_mass_: Topic coherence).

*All Metrics are Percentage (%)*						
Theme	Metric	k-means	LDA	LSA	NMF	BERTopic
Teaching Practice	Accuracy	69.0	78.2	53.0	77.8	66.9
	Cohen’s κ	0.42	0.55	−0.15	0.57	0.34
	Precision	56.3	70.8	0.0	65.3	56.1
	Recall	94.7	75.9	0.0	92.8	73.0
	F1-Score	70.6	73.2	0.0	76.6	63.4
	*U*_mass_	−2.09	−4.65	−2.79	−4.53	−5.47
TA Availability	Accuracy	89.7	83.6	91.0	89.9	60.0
	Cohen’s κ	0.72	0.59	0.78	0.72	−0.04
	Precision	96.7	71.6	84.4	95.2	24.8
	Recall	65.6	68.9	83.4	67.3	20.8
	F1-Score	78.1	70.2	83.9	78.9	22.6
	*U*_mass_	−2.41	−2.24	−2.21	−2.17	−2.00
Q&A	Accuracy	76.4	72.9	50.9	77.3	56.5
	Cohen’s κ	0.38	0.37	0.11	0.45	−0.05
	Precision	80.5	58.9	37.3	70.5	28.4
	Recall	36.6	55.7	73.8	52.4	22.0
	F1-Score	50.3	57.2	49.5	60.1	24.8
	*U*_mass_	−3.01	−2.12	−3.03	−2.53	−2.63
*Overall Performance*						
Average Accuracy		67.6	67.3	47.5	72.5	41.7
Average Cohen’s κ		0.49	0.50	0.23	0.57	0.10
Average *U*_mass_		−2.50	−3.00	−2.68	−3.07	−3.36
Macro F1		66.4	66.9	44.5	71.9	36.9
Micro F1		67.6	67.3	47.5	72.5	41.7
Sample Weighted F1		66.1	67.2	39.7	71.9	39.4

**Table 7:** Performance metrics for TA support topic models (Approach 2).(*U*_mass_: Topic coherence).

*All Metrics are Percentage (%)*						
Theme	Metric	*k*-means	LDA	LSA	NMF	BERTopic
Interactions	Accuracy	73.0	75.2	47.6	79.8	68.9
	Cohen’s κ	0.48	0.50	−0.14	0.60	0.38
	Precision	90.9	75.8	51.2	89.0	74.1
	Recall	56.3	80.2	86.6	71.8	66.2
	F1-Score	69.6	78.0	64.4	79.5	70.0
	*U*_mass_	−2.71	−2.18	−2.62	−2.35	−2.31
Teaching Practice	Accuracy	62.9	65.0	59.1	70.2	62.1
	Cohen’s κ	0.33	0.26	−0.13	0.43	0.25
	Precision	47.6	48.6	4.1	53.6	46.0
	Recall	93.0	60.5	0.9	88.5	69.6
	F1-Score	62.9	53.9	1.5	66.8	55.4
	*U*_mass_	−2.09	−4.65	−2.79	−4.53	−5.47
*Overall Performance*						
Average Accuracy		62.3	64.3	47.6	69.2	59.7
Average Cohen’s κ		0.36	0.34	−0.12	0.46	0.28
Average *U*_mass_		−2.50	−3.00	−2.68	−3.07	−3.36
Macro F1		44.2	43.9	22.0	48.8	41.8
Micro F1		62.3	64.3	47.6	69.2	59.7
Sample Weighted F1		59.3	60.8	35.7	66.1	57.0

#### Peer support dataset.

Quantitative, internal and external performance metrics for the topic modelling of student responses to prompts regarding peer support are summarized in Table 8. In terms of the best per-topic metrics, Approach 2 (A2) to determining ground truth performed consistently better than Approach 1 (A1). However, when considering the average performance metrics across all topics within a model, results were mixed with A1 performing best in LDA and LSA topic models while A2 performing best in the remaining models (*k*-Means, NMF, BERTopic). When comparing the performance of the best approach for each model, the matrix factorization techniques (NMF, LSA) generated the highest scores for both the best, per-topic performance measures and the average metrics across all three topics in a model. In terms of internal performance (measured by *U*_mass_ topic coherence), matrix factorization (LSA) produced the best coherence scores and the smallest variation in coherence (NMF) across the three topics in the models.

**Table 8:** Performance metrics for peer support topic models. (A1: Ground truth Approach 1; A2: Ground truth Approach 2; *U*_mass_: Topic coherence).

		*All Metrics are Percentage (%)*									
Theme	Metric	*k*-means		LDA		LSA		NMF		BERTopic	
		A1	A2	A1	A2	A1	A2	A1	A2	A1	A2
Interactions	Accuracy	51.5	65.2	72.5	61.5	82.2	76.5	79.5	80.9	77.7	83.1
	Cohen’s κ	0.20	0.35	0.18	0.13	0.47	0.47	0.51	0.60	0.48	0.64
	Precision	30.3	54.2	36.0	54.8	59.0	89.9	51.6	80.0	49.1	81.6
	Recall	95.6	91.4	34.7	28.3	57.8	47.3	84.7	70.3	84.7	75.2
	F1-Score	46.0	68.0	35.4	37.3	58.4	62.0	64.1	74.8	62.2	78.2
	*U*_mass_	−5.43	−5.43	−4.30	−4.30	−2.48	−2.48	−3.80	−3.80	−9.26	−9.26
Civility	Accuracy	76.2	73.6	70.5	67.4	82.7	76.7	86.5	80.4	64.6	67.1
	Cohen’s κ	0.23	0.16	0.28	0.21	0.62	0.49	0.68	0.53	0.30	0.35
	Precision	74.0	60.6	46.5	42.4	62.8	56.0	72.1	63.3	42.3	45.4
	Recall	20.6	16.6	49.9	44.5	90.6	79.0	83.1	71.6	79.4	83.4
	F1-Score	32.3	26.0	48.1	43.4	74.2	65.5	77.2	67.2	55.2	58.8
	*U*_mass_	−2.54	−2.54	−2.94	−2.94	−3.24	−3.24	−6.16	−6.16	−8.20	−8.20
Questioning	Accuracy	72.6	88.6	77.5	75.3	84.0	90.4	80.1	90.0	59.8	78.1
	Cohen’s κ	0.46	0.72	0.55	0.51	0.68	0.79	0.60	0.77	0.21	0.39
	Precision	98.8	91.7	78.5	56.8	94.6	77.9	97.3	82.8	98.0	93.3
	Recall	46.7	70.0	76.7	89.7	72.8	97.0	62.7	86.2	21.4	32.9
	F1-Score	63.5	79.4	77.6	69.6	82.3	86.4	76.2	84.5	35.2	48.6
	*U*_mass_	−3.64	−3.64	−6.25	−6.25	−2.63	−2.63	−3.03	−3.03	−6.20	−6.20
*Overall Performance*											
Average Accuracy		50.1	63.7	60.2	52.1	74.4	71.8	73.0	75.6	51.0	64.2
Average Cohen’s κ		0.30	0.42	0.36	0.29	0.60	0.58	0.60	0.63	0.32	0.46
Average *U*_mass_		−3.87	−3.87	−4.50	−4.50	−2.78	−2.78	−4.33	−4.33	−7.89	−7.89
Macro F1		47.3	57.8	53.7	50.1	71.6	71.3	72.5	75.5	50.8	61.9
Micro F1		50.1	63.7	60.2	52.1	74.4	71.8	73.0	75.6	51.0	64.2
Sample Weighted F1		51.1	59.8	60.4	49.2	74.9	70.6	73.9	75.7	46.5	63.5

### Qualitative measures of topic model performance

A qualitative evaluation of topic quality (conducted by a human rather than using statistical topic coherence measures like *U*_mass_) required revisiting the number of topics appropriate for describing the data. While all five topic models independently determined that three topics were optimal, human evaluation via traditional thematic analysis determined that four, three, and five themes for the faculty, TA, and peer support datasets respectively were appropriate (Table 9). For all three datasets, both the traditional analysis and the topic models identified a theme corresponding to *interactions* with students and among students. The *teaching practice* theme also emerged from both traditional analysis and topic models for the faculty and TA support datasets as did the *examples and experience* theme for the faculty support dataset. However, the *hospitality* theme which was prominent in traditional analysis for faculty support failed to emerge as a distinct topic in any of the NLP topic models. Further, the *examples and experience* theme which was identified from traditional analysis of the TA support dataset did not emerge as a distinct theme from the NLP topic models but was instead merged with *teaching practice* by these models. In its place, the topic models split the *interactions* theme into two topics corresponding to TA availability and actual TA Q&A with students. This discrepancy caused the themes and topics to be misaligned in the Approach 2 (A2) assessment of topic model performance. And finally, the topic models absorbed the *preparation* theme into the *civility* theme and the *engagement* theme into the *interactions* theme in the peer support dataset. While these merging events did not compromise the alignment between traditional thematic analysis and corresponding analysis based on topic models, they did reduce the granularity of the results.

**Table 9:** Topics/themes using traditional and topic model analysis.

Dataset	NLP Topics*	Themes	Theme Descriptions (from Traditional Analysis)
Faculty Support	3	4	*Interactions*: interactions with students, active learning, providing feedback to students, facilitating collaborations Examples and Experience: plentiful examples, real-world applications, and resources for learning; quality delivery *Teaching Practice*: reasonable assessment practices, clear and transparent expectations, and well-organized materials and assignments *Hospitality*: kindness, empathy, flexibility, and other elements of care (did not emerge in NLP topic models)
TA Support	3	3	*Interactions*: similar to Faculty Support (split into two topics in NLP topic models) *Examples and Experience*: similar to Faculty Support (did not emerge in NLP topic models) *Teaching Practice*: similar to Faculty Support
Peer Support	3	5	*Questioning*: expectations of other students to ask questions *Interactions:* social interactions and collaborations with other students (merged with *Engagement* in NLP topic models) *Civility:* polite, respectful behavior in the classroom (merged with *Preparation* in NLP topic models) *Engagement:* paying attention and participating in class *Preparation:* coming to class prepared to be productive

*Keywords associated with topics identified by NLP topic models are found in Table 4

### Aggregate performance metrics

**Fig 2:**
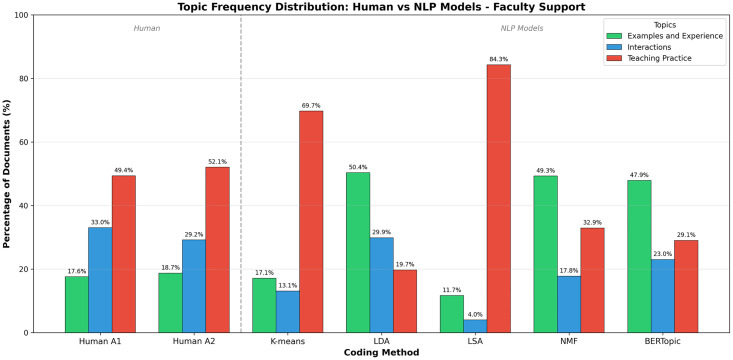
Theme and topic frequencies for faculty support.

**Fig 3:**
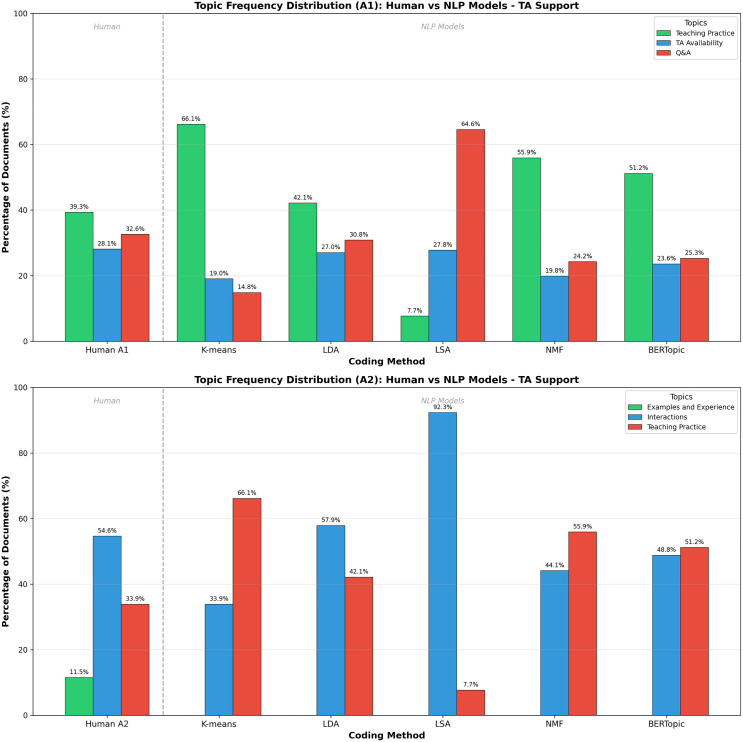
Theme and topic frequencies for TA support.

**Fig 4:**
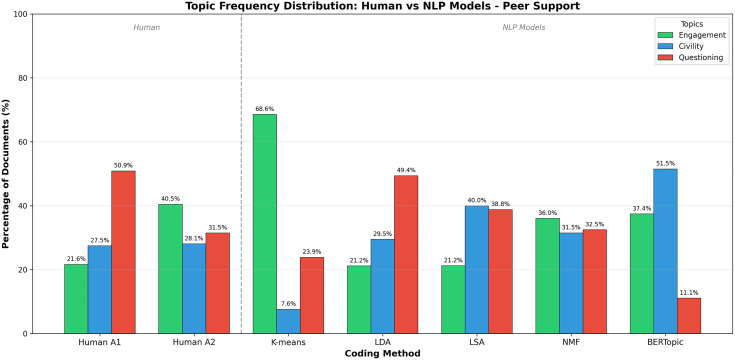
Theme and topic frequencies for peer support.

As an additional measure of topic model performance, topics were ranked by frequency for each model and compared to the rankings determined by traditional (manual) analysis of the three datasets. The rankings determined by each of the five topic models and by traditional analysis are summarized for all three datasets in Table 10. None of the topic models replicated the rankings from traditional thematic analysis for the faculty support dataset (Fig 2), while three of the five topic models replicated rankings for the TA support dataset (Fig 3, Approach A2), and two of the five topic models did so for the peer support dataset (Fig 4, Approach A2).

**Table 10:** Theme rankings.

Dataset		Theme (Rank)*	*k*-means	LDA	LSA	NMF	BERTopic
Faculty Support	A1	Examples & Experience (3)	2	1	2	1	1
		Interactions (2)	3	2	3	3	3
		Teaching Practice (1)	1	3	1	2	2
	A2	Examples & Experience (3)	2	1	2	1	1
		Interactions (2)	3	2	3	3	3
		Teaching Practice (1)	1	3	1	2	2
TA Support	A1	Examples & Experience**	N/A	N/A	N/A	N/A	N/A
		Interactions (2)	1	1	2	2	1
		Teaching Practice (1)	2	2	1	1	2
	A2	Examples & Experience**	N/A	N/A	N/A	N/A	N/A
		Interactions (1)	1	1	2	2	1
		Teaching Practice (2)	2	2	1	1	2
Peer Support	A1	Civility & Preparation (2)	3	2	1	3	1
		Interactions & Engagement (3)	1	3	3	1	2
		Questioning Practice (1)	2	1	2	2	3
	A2	Civility & Preparation (3)	3	2	1	3	1
		Interactions & Engagement (1)	1	3	3	1	2
		Questioning Practice (2)	2	1	2	2	3

*Rank according to Traditional (manual) Thematic Analysis.

**Did not emerge as a distinct theme with topic model analysis.

Rankings indicate theme frequency (1 = most frequent, 2 = second most frequent, 3 = least frequent).

### Interferents in topic model performance

Ambiguity (indicating that a response/document could be reasonably coded as one topic or another topic) was subjectively evaluated for all responses in all three datasets as were multiple topic responses (indicating that a response/document could be reasonable assigned to more than one topic). Results are shown in Table 11. The TA support dataset had the highest rates of ambiguous responses (18.6%), and the faculty support dataset had the highest rate of multiple topic responses (26.5%), while peer support demonstrated the lowest rates overall.

**Table 11:** Ambiguous and multiple topic responses.

	Faculty Support	TA Support	Peer Support
Ambiguous Response	13.7%	18.6%	5.89%
Multiple Topic Response	26.5%	11.7%	6.86%

## Discussion

This study set out to address two research questions regarding how modern NLP-based topic modelling of short texts (STTM) can potentially bridge the gap between machine learning techniques and traditional thematic analysis of short texts associated with education and education research. Five distinct topic modelling techniques (*k*-means clustering, LDA, LSA, NMF, and BERTopic) were applied to three datasets (exploring student expectations for faculty, TA, and peer support). The performance of each topic model was compared to traditional thematic analysis conducted by domain expert using a wide range of performance metrics. Overall (Table 12), no single topic model consistently excelled in extracting topics from the three datasets with high accuracy, reliability (F1-score), topic coherence, and theme ranking. While no one model fits all performance needs, several trends were observed in performance that provide valuable guidance to practitioners who rely on qualitative data analysis to guide their research, teaching, or other pursuits in education.

**Table 12:** Best performing topic models.

Metric Type	Metric	Faculty Support	TA Support	Peer Support
Internal	Single Topic Coherence (*U*_mass_)	BERTopic	BERTopic	LSA
	Average Topic Coherence (*U*_mass_)	NMF	*k*-means	LSA
	Variation in Topic Coherence (*U*_mass_)	NMF	LSA	LSA
External	Single Topic Accuracy	*k*-means	LSA	LSA
	Average Accuracy	*k*-means	NMF	NMF
	Variation in Accuracy	BERTopic	BERTopic	LSA
	Single Topic F1-Score	*k*-means	LSA, NMF	LSA
	Average F1-Score	*k*-means	NMF	NMF
	Variation in F1-Score	BERTopic	LDA	NMF
Aggregate	Topic/Theme Ranking	*k*-means	NMF	NMF
Approach to Developing Ground Truth		A2	A1	A2

### STTM using *k*-means Clustering

In this analysis, *k*-means clustering served as the baseline partitioning and topic modelling method because it treated words as words without regard to semantic relationships or contextual relationships [37]. The *k*-means technique which clusters documents based on distance in a vector space [65], is simple and computationally efficient [66], but is sensitive to initial centroid placement [67] and assumes spherical cluster shapes of similar sizes. When textual data reflect more complex or indirect ideas such as those involving emotion, context, or multiple overlapping topics; *k*-means often splits related responses across clusters or grouped unrelated ones together, thereby ignoring conceptual meaning in the process [68]. Given these limitations, it is surprising how well *k*-means did in replicating the perspective and assessments of a domain expert (Approach A2) with the faculty support dataset. A closer look at the vocabulary associated with each of the three themes (*examples and experience*, *interactions*, *teaching practice*) in this dataset gives some important insight into why this is the case. The words and vocabulary that students use in describing their preferences for how faculty should support them within each of these three themes are very distinct. The words “office hours,” “available,” “email” almost exclusively belong to the *interactions* theme while the words “example,” “real world,” “experience” uniquely signal *examples and experience* and the words “teach,” “explain,” “lecture,” “resources”, and “organized” refer to *teaching practice*. These distinct choices of words form tight, well-separated clusters in word embedding (TF-IDF) space, which is exactly what *k*-means clustering optimizes. Furthermore, the fact that many of the faculty support responses students provided are longer (with an average length of 10 words) than those provided for TA and peer support provides k-means with more lexical information to work with, further supporting its ability to excel in classifying the faculty support dataset. Another more subtle performance advantage to *k*-means for this dataset is its clear bias toward the *teaching practice* theme, assigning approximately half of student responses to this theme. Since *teaching practice* is the majority class, this bias works in favor of *k*-means, improving correct topic assignments by chance. Such bias is coincidental and cannot be relied upon to manifest in every dataset. Nevertheless, the success of the *k*-means topic model on the faculty support dataset (and corresponding failure on the peer support dataset) illustrates a key point regarding the selection of topic models: more sophisticated models are not always better than simpler models. Instead, more complex methods should be used only when topics or themes share substantial vocabulary and require decomposition (NMF, LSA), probabilistic analysis (LDA), or reliance on pretrained language models (BERTopic) to separate.

### STTM using latent dirichlet allocation (LDA)

LDA, a widely used topic modelling algorithm, represents each document as a mixture of topics and each topic as a mixture of words. To do so, however, LDA relies heavily on the same words occurring in multiple documents, making short texts prone to inaccurate topic assignments [69,70]. While this limitation is likely to have detrimentally affected the performance of LDA modelling relative to other topic models in this analysis, other factors must also be contributing to LDA performance, since the dataset with the shortest texts (peer support) produced the best results among all the LDA topic models. One possibility is that the improved performance of LDA in the peer support dataset resulted from the fact that fewer student responses were ambiguous (Table 10) compared to the TA support and faculty support datasets. Since LDA was constrained in this analysis to assign only a single topic to each document in each dataset and it does not consider context, word sequence, word order, or any other semantic information in topic assignments, topic selection is likely to be random in ambiguous cases, leading to errors in topic assignment compared to domain expert assessment. A domain expert is more able to assess subtle differences in the choice of language (i.e., the “noise” in the data) necessary to determine the dominance of a single topic when ambiguous language is in use. While the lack of robustness in the face of ambiguity is a weakness of LDA, it also serves as a guide to topic model selection: when ambiguity in documents is frequent, LDA is to be avoided.

If ambiguity were the only factor contributing to the poor performance of LDA, however, a decline in accuracy and F1-score from the peer support dataset (low ambiguity) to the faculty and TA support datasets (higher ambiguity) would be expected. Since this is not the case, other factors must be at play which contribute to LDA’s poor performance relative to other topic models. Prior research has noted that the reliability and validity of LDA-derived topics can be limited when short responses provide sparse word co-occurrence information [61]. In this comparative analysis, however, word co-occurrence is particularly frequent in the peer support dataset where the words “ask” and “questions” (corresponding to the *questioning* theme) occur in 60.6% of the documents assigned to this theme which, not surprisingly, results in superior model reliability (F1-scores of 77.6% and 69.6% and 77.5% and 75.3% accuracy for Approach 1 and Approach 2 respectively) compared to the LDA F1-scores and accuracy for the remaining two themes. This result underscores the value of LDA in datasets where the same words are frequently used to express similar ideas.

### STTM using matrix factorization (LSA and NMF)

Unlike LDA, matrix factorization considers relationships between words by grouping words that often appear together in documents [71]. This approach goes beyond treating documents as mere sets of words and provides some elementary insight into the latent semantic structure (meaning) of what is being said with those words (i.e., their context). Consideration of basic semantics is a major contributor to the fact that 63% of the best performers in terms of internal and external performance metrics (Table 11) are matrix factorization methods (NMF or LSA).

#### Latent semantic analysis.

Despite considering semantic meaning more so than *k*-means and LDA, LSA still struggles with words (or groups of words) that have multiple meanings [72]. This vulnerability was demonstrated in the faculty support dataset in the *examples and experience* theme where interrater reliability (Cohen’s κ) was worse than that expected by chance (indicated by negative scores). Students often used the word examples to express different ideas. Consider the following three documents:

Go through lots of examples, and when they do, explain your thought process.
Have clear, focused lectures with supplementary materials such as lecture notes and practice problems available.
To give abstract examples for topics before going to examples.

While all three documents refer to opportunities for practice or examples, the first is the only one that is pedagogically related to the *examples and experience* theme, where teachers expose their thought processes around problem solving and gradually reduce scaffolding until students can solve similar problems on their own. The remaining two documents refer to *teaching practice* with regard to how and what teachers present in lecture and how they supplement their lectures with readily available, external resources. In classifying the faculty support dataset, the LSA model also performs particularly poorly in classifying documents as *interactions*, producing a large number of false negatives (missed responses) as evidenced by recall scores of 12.1% and 13.7% (Table 5). This poor recall is a direct consequence of how LSA constructs its topic dimensions using singular value decomposition (SVD). SVD requires its components to be mathematically orthogonal, which forces the weight of each word to be distributed across multiple topic dimensions rather than concentrated across a single dimension. The words “office” and “hour” which together appear in 41.8% of interactions documents but only 4% of documents belonging to other themes are the strongest indicators for the interactions theme. However, these words appear so frequently in the overall corpus that they are captured by the first SVD component which represents the maximum variance in the data and also corresponds to the *teaching practice* topic. As a result, LSA assigns them substantial weight on both the *teaching practice* dimension and the i*nteractions* dimension. This near-equal loading means that documents containing “office hours” are only marginally more likely to be assigned to *interactions* than to *teaching practice*, and in practice the vast majority are absorbed into the larger *teaching practice* cluster where of the 553 ground truth interactions documents, LSA misclassified 474 as *teaching practice*. In contrast, NMF does not experience this problem because its non-negativity constraint enforces sparsity. Each word concentrates its weight on the single topic where it contributes most which causes “office” and “hour” to load exclusively on the *interactions* dimension with zero weight on all other components, allowing NMF to cleanly separate documents containing these words and produce recall scores substantially higher than LSA for this theme. On the plus side, poor recall scores that require such detailed attention are typically confined to a single topic which while not ideal, may limit the impact of this LSA behavior.

#### Non-negative matrix factorization.

NMF topic models decompose the document-term matrix into two non-negative matrices – one representing topics and another representing word/term weights [21]. Previous research has often suggested that this decomposition process produces more coherent topics than other topic modelling techniques [12,34]. However, in this comparative analysis, the statistical measures used to evaluate topic coherence (*U*_mass_) only partially supported existing literature in that average coherence scores were worse than two other topic models in two of the three datasets (peer support and TA support). A qualitative (human) evaluation of topic quality and coherence using the keywords in Table 4 for NMF, however, negates these poor topic coherence scores. For example, the keywords “ask, question, answer, think, afraid” clearly point to not being afraid to asking and answering thoughtful questions, aligning with the pedagogically theme of *questioning* practice. The same is true for keywords “talk, participate, distract, respectful, quiet” relating to civil behavior and aligning with the *civility* theme and “study, group, help, willing, discussion” relating to collaboration among peers and aligning with the *interactions* theme. The fact that qualitative (human) evaluation indicates that NMF generated topics with strong topic coherence while statistical coherence does not strongly suggests that it is not the sparsity or brevity of the documents that is the issue but rather sensitivity to noise words in the documents that limits an accurate statistical perspective on coherence. This contradiction also underscores the need to include qualitative assessment of topic model performance rather than relying exclusively on quantitative metrics.

Despite the cautionary tales regarding the confounding influence of multiple meanings of key words (LSA) and noise words in the data (NMF), the matrix factorization methods dominated the performance of the topic models in this dataset (Table 12). While superior performance can be largely attributed to the consideration of semantics and meaning among words by matrix factorization methods, it leaves open the question of why BERTopic which considers semantics at a much more sophisticated level did not produce even better results.

### STTM using neural networks (BERTopic)

While LSA and NMF rely on patterns determined by the presence of the same combinations of words in different documents to establish co-occurrence and semantic meaning, BERTopic goes a step further by taking into account words surrounding other words (i.e., contextual embeddings) to determine meaning and using clustering techniques to organize and capture these deeper semantic relationships within text [73]. In this analysis, however, BERTopic failed to deliver. But BERTopic’s performance deficits herein do not spell doom for this popular and sophisticated topic model. Rather, they underscore the need to optimize BERTopic’s capabilities by selecting appropriate scaffolding for the corresponding topic models. In this analysis, *k*-means clustering was substituted for HDBSCAN in BERTopic’s pipeline to ensure reproducibility and to force the number of discovered topics to three. While this choice was necessary for a fair, one-for-one comparison among the five topic models, this substitution minimized three essential capabilities available in BERTopic model design. First, HDBSCAN (a hierarchical clustering method) identifies outliers, allowing documents that do not fit naturally into any cluster to be set aside rather than forcing them into the nearest cluster regardless of how far away in semantic space that nearest cluster resides. Allowing discovery of outliers is important for datasets containing documents with multiple topics or ambiguous topic assignments, because such documents occupy boundary regions between clusters and introduce noise and errors when forced into a single topic. In this analysis, the faculty support and TA support datasets had much higher proportions of multiple topic and ambiguous documents, leading directly to a degradation in performance from peer support results to those for faculty and TA support. Second, *k*-means assumes clusters are spherical and of similar size. In BERTopic, however, the dimensionality reduction step implemented in the modelling pipeline prior to clustering (UMAP) intentionally produces irregular, density-varying cluster shapes that only HDBSCAN is designed to detect. In effect, replacing HDBSCAN with *k*-means discards the density structure that UMAP was specifically designed to preserve, hobbling its ability to leverage semantic meaning into accurate and interpretable topics that align with relevant themes in the data.

### The role of vectorization in topic discovery

An important consideration that cuts across the model-by-model comparisons above is the role of text vectorization in shaping topic discovery. In this analysis, LDA operated on raw word counts (bag-of-words), k-means, LSA, and NMF operated on TF-IDF representations, and BERTopic operated on contextual embeddings from a pretrained Sentence-BERT model. These are not interchangeable preprocessing steps; each one determines what information the topic model has access to and, consequently, what patterns it can detect. When comparing LDA to NMF or LSA, for example, the observed performance differences reflect not only the mathematical properties of the models themselves but also the advantage that TF-IDF confers over bag-of-words in short-text settings. TF-IDF amplifies rare, discriminative words such as “office” and “hours” while suppressing common words such as “class” and “help” that appear across all three themes, giving the model stronger signal from documents that may contain only six to ten words. LDA, by contrast, treats every word occurrence equally, leaving it with less discriminative information in sparse, short documents. This confound is important to acknowledge: the consistently stronger performance of TF-IDF-based models relative to LDA across all three datasets (Tables 4-9) cannot be attributed to the topic model alone.

Contextual embeddings, as used in BERTopic, encode an additional layer of semantic information by representing documents based on how and where words appear relative to one another rather than simply which words are present. This feature is most valuable when themes share substantial vocabulary and can only be distinguished by meaning rather than by the presence or absence of specific words. However, when the vocabulary associated with each theme is already distinct, as in the faculty support dataset, the additional semantic information offers little practical benefit over TF-IDF because the themes are already well-separated in frequency-based vector space. For practitioners, these observations suggest that vectorization should be treated as a deliberate design decision rather than a default byproduct of model selection, and that the expected degree of lexical overlap among themes in a given dataset should inform that decision alongside the choice of topic model itself.

### Ground truth (methodological) considerations

To address our second research question (RQ2), whether there are significant differences in machine-led vs. human-led application of STTMs that indicate bias, this study used two approaches for calculating external performance metrics which compared the topics assigned by a topic model to a “ground truth” to assess accuracy and reliability of the model. The first approach (A1) was led by the topic model (machine-led) where the top words from each topic and each model were distilled into a list of keywords that enabled a domain expert to code the data manually. The second approach (A2) was machine-supported (and human-led) where a domain expert coded the data independently using only the optimal number of topics determined by the topic models as a guide. Our findings indicated that Approach 2 led to substantially better performance for most models used to analyze the faculty and peer support datasets. This result suggests that topic models can indeed directly identify pedagogically relevant themes and, while unexpected, adds to existing literature that advocates for topic models to replace a great deal of the tedious and time consuming work involved in the qualitative analysis of text-based data. However, the fact that Approach 2 fails altogether in the analysis of TA support data tells another cautionary tale about over-reliance on machine learning for the analysis of language. In the case of the TA support data, the topic models produced topics that were misaligned with the themes identified by the domain expert in Approach 2, leading to very poor performance across the board. While the topic models “thought” that the three topics in the data were *Q&A*, *availability*, and *teaching* practice (which absorbed *examples and experience* into overall teaching practice), a domain expert thought differently, assigning themes of *interactions* (which combined the Q&A and availability topics), *examples and experience*, and *teaching practice* to the data. Thus, while topic models can indeed replicate themes assigned by domain experts using traditional analysis methods, topic-to-theme alignment is not guaranteed and must be supervised by a human being to avoid the largely erroneous results produced for TA support in this analysis.

### Implications

Although the results and discussion to this point have addressed the two research questions of this comparative analysis, *RQ1 regarding topic model performance on education datasets and RQ2 regarding differences between machine-led and human-led approaches* to establishing ground truth, the overall goal of our work is to provide actionable recommendations to practitioners (instructors, TAs, researchers, and other stakeholders) for integrating NLP-based topic models into their work at all levels of education. While neither the comparative analysis conducted herein nor the previous literature relevant to this analysis can provide a comprehensive set of recommendations for using NLP effectively, some key insights have emerged (Table 13). It is important to note that all topic models considered in this comparative analysis are baseline models, using default parameters and constructed in such a way that one-for-one comparison among the models is possible with minimal bias (caused by varying levels of optimization). The resulting recommendations are therefore starting points for analysis; further optimization of the chosen topic model will only improve the value and the performance of the results.

**Table 13:** Recommendations for using topic models in education practice.

I have a dataset (corpus) of documents and….	Recommendation
I have no idea what they are telling me.	Process the documents with a simple topic model (*k*-means, LDA) using the optimal number of topics determined by the elbow method as well as fixed numbers of topics on either side of this optimal number to explore the data using the top *n* words for each topic or word clouds.
I want to quickly identify the low-hanging fruit in my dataset so I can spend my time focusing on the documents that are more difficult to interpret.	Process the documents with LDA using the optimal number of topics determined by the elbow method. Generate word clouds of the results and seek out frequent word co-occurrences in the data (indicated by large font in the word clouds). Use these word co-occurrences to fast-track coding of documents that contain them.
I have looked at my data and noticed that students/respondents are using distinct (non-overlapping) vocabularies to express different ideas.	Process the documents with *k*-means using the optimal number of topics determined by the elbow method as well as fixed numbers of topics on either side of this optimal number to enable identification of dominant topics in the data. Align the topics with themes relevant to the research question or task at hand.
While there seem to be a few distinct patterns in the data, there are subtle differences within patterns that I am interested in diving into.	Use BERTopic with UMAP dimensionality reduction and HDBSCAN clustering to break the data down into as many natural clusters as are relevant. Combine clusters via human evaluation as necessary to find themes relevant to the research question or task at hand.
Students/respondents are using similar but not the same words to express the same ideas.	Process the data with a matrix factorization method (e.g., LSA, NMF) using the optimal number of topics determined by the elbow method to gain a basic idea of semantic similarity across different documents. Advance to BERTopic if granularity is insufficient to address the task at hand.
Analysis of my data is worthless unless resulting topics or groups of documents have clear semantic meaning.	Process the data using NMF and the optimal number of topics determined by the elbow method as well as fixed numbers of topics on either side of this optimal number. Adjust NMF parameters to optimize both statistical coherence (e.g., *U*_mass_) *and* qualitative coherence (human evaluation of top words corresponding to each topic).
Although my documents tend to use the same sets of words frequently, I need to identify those documents that are ambiguous so I can have multiple human evaluators figure out what’s going on with them.	Process the data using LDA and the optimal number of topics determined by the elbow method. Filter out documents where one topic probability stands out (is much higher) than the others. Consider the remaining documents ambiguous.
I know my documents have outliers that are pretty much not interpretable or irrelevant and I want to filter those out before proceeding with the more meaningful documents.	Use BERTopic with UMAP dimensionality reduction and HDBSCAN clustering to identify outliers. Manually evaluate outliers for different types of anomalous behavior and eliminate documents from further analysis as desired.
My documents are very short and use a wide range of words (large vocabulary)	Avoid LDA. Use a matrix factorization method (e.g.,. LSA, NMF) to gain a basic understanding of how semantic meaning differs among different topics. Advance to BERTopic if results do not provide sufficient insight or granularity.

(Note: this study used k-means within BERTopic to fix k = 3 for cross-model comparison.)

Regardless of topic model type or optimization approach, this study has added to the existing literature in cautioning against fully automated analysis of text-based data when a high degree of rigor or performance is required. In these situations, which are very common in education and education research, manual (human) intervention is not only desirable but required to make sure that topic modelling does not lead the analysis astray and into unacceptably erroneous conclusions. While STTM is clearly a useful tool in thematic (and other related qualitative) analyses of text, it is in no way a magic bullet replacement for domain expert assessment.

### Limitations

While this study provides a comprehensive comparative analysis of basic topic modelling techniques, it is not without limitations. Most importantly, the scope of the dataset was confined to a single institution, a specific type of educational data (student feedback), and a particular context (engineering courses). These constraints could limit the generalizability of the findings to STTM in other data, settings, and contexts. To broaden the relevance of the findings in this study, guidelines for selecting the best topic model and model parameters are outlined in the Implications section of this manuscript and rely on data characteristics that are largely independent of the domain and context in which the data are collected.

Furthermore, our dataset, while sizable, might still be considered moderate (between one and two thousand responses per dataset/corpus after preprocessing) by big data standards. While a larger dataset is likely to improve performance, however, it is unlikely to change the nature of the results (number of themes, meaning of themes, etc.). Theme assignment and evaluation in this study was also limited to a single theme per document, which ignored the possibility that multiple themes were present in some documents. Multiple topic documents can deflate topic coherence scores by introducing words from secondary topics into the assigned topic. Fortunately, these deflationary effects can reasonably be expected to be consistent across models, thereby having minimal impact on the relative performance of one topic model compared to others.

Another potential bias in the results could emerge from assuming that theme assignments by a human domain expert were the gold standard (i.e., ground truth). This source of error was mitigated by ensuring that multiple domain experts came to the same conclusions regarding theme assignments as indicated by more than adequate Cohen’s kappa (i.e., interrater reliability). Interpretation of model results may also be biased by using predominantly quantitative performance metrics to compare models (accuracy, precision, recall, F1-scores, statistical topic coherence). Future work should consider an expanded and complementary comparative study that focuses on qualitative assessments of model performance including but not limited to multiple qualitative assessments of topic quality, topic diversity, and topic interpretability measures.

Finally, in terms of model selection, this analysis also focused on five popular but basic topic models, largely ignoring the wide range of variations developed for these models that have been demonstrated in the literature. Some of these variants may be better aligned with the type of data in this study and could substantially improve the performance over the basic model in addition to providing more granularity and more topics to describe the data. However, since the goal of this study was to develop broad guidelines that provide a starting point for instructors, researchers, and other practitioners in education to use NLP in their work, further examination of variants or advanced NLP topic models fell outside the scope of this study.

## Conclusions

A comparative analysis of five common topic models has been conducted in the analysis of three datasets associated with teaching practice and education research. The topic models were constructed to enable a one-for-one comparison among models by using similar pipeline design principles as well as default model parameters. Performance results showed that simpler models can be the best models in certain situations (e.g., *k*-means analysis when distinctly different vocabularies are used to express different ideas) and that unadorned matrix factorization methods performed consistently and reliably. This study also underscored the fact that advanced models based on neural networks or more sophisticated algorithms (e.g., BERTopic) are valuable but only when their modelling pipelines are structured to make the most of their strengths. For this reason as well as for the increased computing power and processing time incurred by these more advanced models, more traditional topic models are often the best place to start. This study also showed that even traditional topic models, which are often considered simplistic or outdated, are capable of identifying topics that align well with conceptually relevant themes with minimal human intervention. Regardless of topic model, however, this study confirms that to maintain rigor, accuracy, and reliability of qualitative analysis, strategic intervention of a human being or domain expert in machine-driven topic modelling and classification is essential. To this end, future work will focus on evaluating a wider range of strategies for human intervention in qualitative analysis of text as well as comparing the performance of topic models beyond their basic implementations by optimizing the models for specific data characteristics and tasks.
